# Cobalt-Assisted Morphology and Assembly Control of Co-Doped ZnO Nanoparticles

**DOI:** 10.3390/nano8040249

**Published:** 2018-04-17

**Authors:** Xianying Han, Sebastian Wahl, Patrícia A. Russo, Nicola Pinna

**Affiliations:** Institut für Chemie and IRIS Adlershof, Humboldt-Universität zu Berlin, Brook-Taylor-Str. 2, 12489 Berlin, Germany; hanxiany@hu-berlin.de (X.H.); sebastian.wahl@chemie.hu-berlin.de (S.W.)

**Keywords:** non-aqueous sol-gel, benzyl alcohol, doping, zinc oxide

## Abstract

The morphology of metal oxide nanostructures influences the response of the materials in a given application. In addition to changing the composition, doping can also modify the morphology of a host nanomaterial. Herein, we determine the effect of dopant concentration, reaction temperature, and reaction time on the morphology and assembly of Co_x_Zn_1−x_O nanoparticles synthesized through non-aqueous sol-gel in benzyl alcohol. With the increase of the atom % of cobalt incorporated from 0 to 15, the shape of the nanoparticles changes from near spherical, to irregular, and finally to triangular. The tendency of the particles to assemble increases in the same direction, with Co_0.05_Zn_0.95_O consisting of non-assembled particles, whereas Co_0.15_Zn_0.85_O consists of triangular nanoparticles forming spherical structures. The morphology and assembly process are also sensitive to the reaction temperature. The assembly process is found to occur during the nucleation or the early stages of particle growth. The cobalt ions promote the change in the shape during the growth stage of the nanoparticles.

## 1. Introduction

Metal oxide nanostructures find application in a broad range of fields that include catalysis [1], energy storage and conversion [2,3,4], sensing [5], and medicine [6]. Their functionalities derive from the unique electronic, optical, and magnetic characteristics, as well as from surface lattice distortions/defects and surface reactivity arising at the nanoscale. As these properties depend on the chemical composition, doping is a valuable strategy for modulating or creating new properties of a host nanomaterial in a controlled manner [7]. Additionally, for a given composition, most properties show a strong dependence on the size and morphology of the nanostructures [8]. For example, the catalytic activity of nanoparticles depends on the type of facets exposed at the surface (i.e., their morphology), as different facets have different energies and therefore different chemical activity [9,10].

Non-aqueous sol-gel synthesis approaches in organic solvents have been successfully applied to the fabrication of a large variety of nanostructures from pure inorganic, to organic-inorganic hybrid materials [11,12,13]. They provide good control over the composition, size, shape, assembly, and crystallinity of nanomaterials, features that are influenced by factors such as the type and reactivity of the precursors, solvent, temperature, reaction time, or the presence of surfactants. In non-aqueous sol-gel processes, the oxygen-supplying species for the formation of metal oxides are generally either the solvent or the precursors. The moderate reactivity of the precursors in organic media leads to slow reaction rates in the formation of metal oxides, resulting in the production of highly crystalline materials at low/moderate reaction temperatures. This is particularly useful for synthesizing doped metal oxides.

Surfactant molecules containing carboxylic acid, phosphoric acid, or amine moieties are commonly employed for controlling the morphology of nanomaterials [14]. These molecules may preferentially bind to specific crystallographic facets during the growth of the nanoparticles and promote the growth in certain directions, which results in the formation of anisotropic nanoparticles of uniform size and shape. In addition, capping molecules can direct the assembly of particles through intermolecular forces such as hydrophobic interactions, hydrogen bonding, molecular dipole interactions or π–π interactions [12,15].

Dopants or impurities can also have a significant impact on the morphology of host nanomaterials, as the adsorption of metal cations onto the facets of nanoparticles may change the growth rates along different directions [16,17]. Although the effect of dopants or impurities on the morphology of nanomaterials has been less investigated than that of surfactants, several studies have revealed that the presence of small ionic species in the reaction mixture can help direct the crystal growth of metals [18], metal chalcogenides [19,20], and metal oxide nanostructures [17,21,22,23]. Bose et al. [20] reported the modification of the morphology of ZnSe nanocrystals upon doping with Mn, from hemispherical-like nanostructures of the undoped ZnSe to spherical nanocrystals for Mn doped. A similar shape change effect with doping was observed for Nb-doped TiO_2_ [23]. TiO_2_ formed platelet nanoparticles, which evolved from platelets to peanut-like 1D nanorods with the addition of Nb. Yang et al. [17] reported morphology and crystal phase changes for Mg doped ZnO with increasing the dopant content. Tetrapods, ultrathin nanowires and irregular nanoparticles were obtained for different Mg contents. The doping of ZnO with Cd^2+^, Mn^2+^, and Ni^2+^ was also studied. Consequently, the addition of appropriate foreign ions to the synthesis of certain nanomaterials, even if they are not incorporated into the final product (i.e., doping), has been recently considered as another strategy for controlling the morphology of nanomaterials [16].

Zinc oxide is used in a wide variety of applications such as catalysis, sensing, and optoelectronic devices [24,25]. Doping with transition-metals such as Co creates new properties (e.g., magnetic) and modifies its optical and catalytic behavior, extending the range of applications [26,27]. ZnO and doped ZnO with various morphologies have been successfully synthesized in organic solvents [17,28,29,30,31,32]. In particular, the synthesis in benzyl alcohol is appealing for producing crystalline ZnO and transition-metal-doped ZnO nanomaterials with fairly uniform size and morphologies at low temperature without the use of surfactants, and for achieving high levels of substitutional doping of Co^2+^ in the ZnO wurtzite structure without the formation of segregated phases [29,30,31]. Herein, we report the effect of cobalt doping on the morphology and aggregation behavior of ZnO nanoparticles synthesized by non-aqueous sol-gel in benzyl alcohol. The shape of the nanoparticles changed from near spherical to triangular with the variation of the Co atom % in the ZnO host from 0 to 15. In addition, for the highest Co content, the particles assemble into spherical nanostructures. The effects are dependent on the reaction temperature.

## 2. Materials and Methods

### 2.1. Synthesis of Co-Doped ZnO

The syntheses of the Co-doped ZnO materials were performed as follows: 1 mmol of zinc acetate (Aldrich, Munich, Germany, 99.99%) and 0.1, 0.2, or 0.3 mmol of cobalt(II) acetate (Aldrich, 99.995%) were added to 5 mL of benzyl alcohol (Aldrich, 99.8%) in a 10 mL microwave glass vial, under argon. The mixture was sealed with a silicone cap under Ar. Subsequently, the suspension was heated in a microwave reactor (Anton Paar Monowave 300, Graz, Austria) at 170 °C for 5 min (with a 50 s heating ramp to reach the final temperature), and finally rapidly cooled down with compressed air. The reaction temperature was controlled with a fiber-optic temperature probe inserted inside the reaction vial. The solid products were collected by centrifugation, washed three times with ethanol, and dried at 70 °C overnight. The samples obtained from the reaction mixtures containing 0.1, 0.2, and 0.3 mmol of cobalt acetate are denoted Co_0.05_Zn_0.95_O, Co_0.09_Zn_0.91_O, and Co_0.15_Zn_0.85_O, respectively (based on the Co atom % determined by Energy dispersive X-ray spectroscopy (EDX) analysis). The synthesis of Co_0.15_Zn_0.85_O was also performed at 170 °C for different time periods. The procedure described above was repeated, except that the reaction was stopped and quickly cooled down under compressed air flow after being kept at 170 °C for 30 s, 45 s, 60 s, 90 s, and 150 s. The solid products were collected by centrifugation, washed three times with ethanol, dried at 70 °C, and characterized. Additionally, the material denoted Co_0.15_Zn_0.85_O was synthesized at 180 °C and 190 °C. The undoped ZnO sample was synthesized in the same way in the absence of cobalt precursor and using 2 mmol of zinc acetate precursor.

### 2.2. Characterization

Powder X-ray diffraction (XRD) patterns were recorded with a STOE MP diffractometer (STOE, Darmstadt, Germany) in transmission configuration using Cu Kα radiation (λ = 0.1541 nm). The measurements were performed in the 2θ range 5–90° with a step size of 0.5°. Transmission electron microscopy (TEM) images were acquired on a Philips CM 200 microscope (FEI, Hillsboro, OR, USA) at 200 kV. For determining the size of the nanoparticles by TEM, the size of ca. 50 nanoparticles was measured on several TEM images. Energy dispersive X-ray spectroscopy (EDX) analysis were performed using an EDAX SDD detector (EDAX Inc., Mahwah, NJ, USA) coupled to the TEM. Diffuse reflectance ultraviolet-visible spectra were collected with a Perkin Elmer LAMBDA 950 Ultraviolet-visible (UV-vis) spectrophotometer (Perkin Elmer, Waltham, MA, USA) equipped with a 150 mm integration sphere using BaSO_4_ as a reference in the wavelength range of 200–800 nm. Fourier transformed infrared (FTIR) spectra were measured on a Thermo Scientific Nicolet iS5 spectrometer (Thermo Fisher Scientific, Waltham, MA, USA) in the wavenumber range of 4000–400 cm^−1^ (4 cm^−1^ resolution), using pellets of the solid diluted in KBr. Carbon elemental analyses were performed on a HEKAtech Euro EA CHNSO Elemental analyzer (HEKAtech GmbH, Wegberg, Germany).

## 3. Results and Discussion

**Figure 1:**
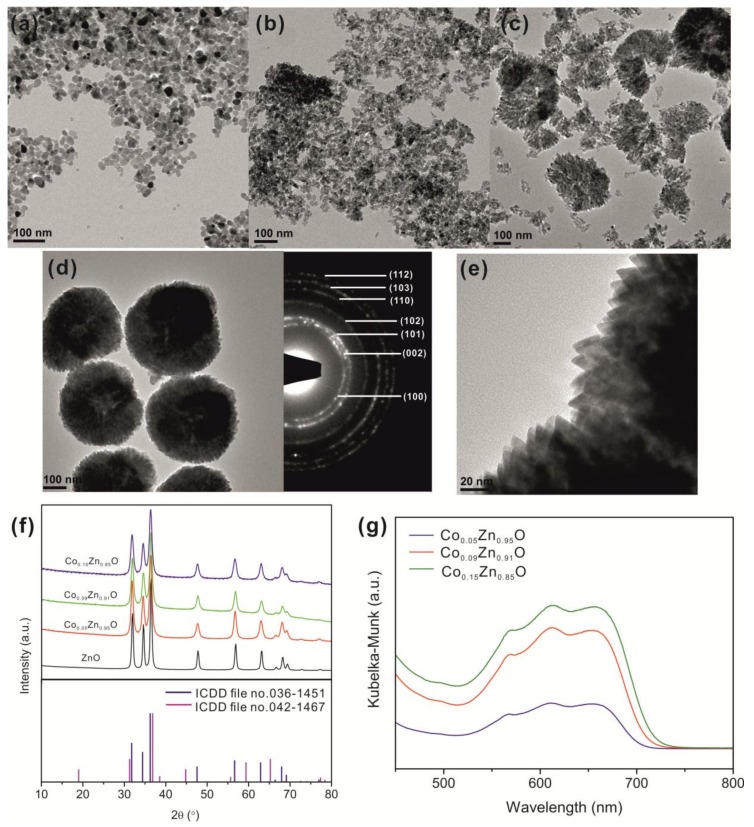
TEM images of (**a**) ZnO, Co-doped ZnO with (**b**) 5, (**c**) 9, and (**d**,**e**) 15 atom % of cobalt (as determined by EDX analysis); right part of (**d**) shows the selected area electron diffraction (SAED) pattern of Co_0.15_Zn_0.85_O nanostructures; (**f**) X-ray diffraction patterns of the ZnO and Co-doped ZnO materials (vertical lines correspond to reference patterns: blue-ZnO, pink-Co_3_O_4_; (**g**) diffuse reflectance UV-vis spectra of the Co-doped ZnO nanomaterials.

The reaction between zinc acetate and benzyl alcohol at temperatures around 170 °C produces crystalline wurtzite zinc oxide nanoparticles with size between 10 and 20 nm and quasi-spherical morphology (Figure 1a). The process involves an esterification reaction that starts with the nucleophilic attack of the oxygen of the alcohol to the carbon of the carbonyl group of the acetate ligand of the metal precursor and leads to the formation of benzyl acetate and hydroxylated zinc species [33]. The latter constitute the monomers for the formation of the zinc oxide, which occurs through condensation reactions of the hydroxylated zinc species with release of water. The same reaction mechanism allows the incorporation of transition-metals such as Co, Fe, Ni or Mn into the host ZnO structure [29,31].

Figure 1b–e shows the TEM images of the Co-doped ZnO nanostructures containing different amounts of cobalt, synthesized by reacting zinc and cobalt acetates with benzyl alcohol at 170 °C under microwave irradiation. The presence of cobalt in the products was confirmed by EDX analysis (Figure S1), and the amounts of dopant measured are 5, 9, and 15 atom % for Co_0.05_Zn_0.95_O, Co_0.09_Zn_0.91_O, and Co_0.15_Zn_0.85_O, respectively. The cobalt contents in the final products are slightly lower than the nominal amounts, likely due to the lower rate of cobalt incorporation compared to the growth rate of the ZnO host particles. Nevertheless, as the Co^2+^ and Zn^2+^ ions have similar sizes in the tetrahedral environment of the oxide, and are both borderline Lewis acids and therefore have similar reactivity, a high amount of 15 atom % of Co was introduced in the ZnO. The XRD patterns of the three doped ZnO materials show reflections arising exclusively from the hexagonal lattice of the host ZnO (Figure 1f), and no significant shifts of the diffraction angles are observed with the increase of the cobalt content. The average crystallite sizes, calculated from the (101) reflections with the Scherrer equation, are 17, 11, 12, and 11 nm for ZnO, Co_0.05_Zn_0.95_O, Co_0.09_Zn_0.91_O, and Co_0.15_Zn_0.85_O, respectively, which are consistent with the sizes of the primary nanoparticles measured from the TEM images. Shifting of the reflections to higher angles (shifts up to 0.1° 2θ) with increasing the cobalt atom % in the ZnO structure, which are indicative of small lattice contractions, have been observed by some authors [34] while others, as in this work, have not detected a clear trend [29]. This behavior is associated with the close proximity of the sizes of the Co^2+^ (0.58 Å) and Zn^2+^ (0.60 Å) ions in tetrahedral coordination, and, consequently, the substitutional doping of Co^2+^ in the tetrahedral Zn^2+^ sites does not cause drastic alterations of the lattice parameters. It has been extensively reported in the literature that the substitution of Co^2+^ at the tetrahedral Zn^2+^ sites in the wurtzite structure originates a broad band in the visible region of the UV-vis spectra of Co-doped ZnO materials [34,35,36]. This band is made of three contributions that arise from d–d transitions. Figure 1g displays the d–d transitions region of the diffuse reflectance UV-vis spectra of the Co-doped ZnO nanostructures with different amounts of cobalt. The spectra show the typical three bands at 567 nm, 610 nm and 657 nm, due to the ^4^A_2_(F) → ^2^E(G), ^4^A_2_(F) → ^4^T_1_(P), and ^4^A_2_(F) → ^2^A_1_(G) transitions, which indicate the presence of Co^2+^ in the tetrahedral environment of the oxide, and that substitutional doping of the Co^2+^ for Zn^2+^ occurred for all the materials.

The TEM images of the doped samples reveal significant changes in the morphology and assembly of the nanoparticles as the amount of dopant increases from 5 to 15 atom %. The Co_0.05_Zn_0.95_O material consists of irregularly shaped nanoparticles (Figure 1b), whereas Co_0.09_Zn_0.91_O contains a mixture of irregular particles and faceted triangular nanoparticles. In addition, some of the latter are assembled forming half-spherical arrangements (Figure 1c). As the Co. atom % increases to 15, the material consists mostly of triangular nanoparticles (of ca. 10–15 nm) assembled into spherical structures of ca. 350 nm in size (Figure 1d,e). The selected area electron diffraction (SAED) pattern of the assembled structures shows the reflections of the ZnO hexagonal lattice. The nanoparticles are cristallographically randomly oriented in these assemblies. These results suggest that the Co^2+^ ions promote the formation of triangular nanoparticles and their assembly into large structures. The change in the morphology of the nanoparticles can be explained considering that adsorption of the dopant species on the surface of the host during growth is a crucial step of the doping process [37,38]. The adsorption energies and residence times depend on the crystallographic surfaces to which the dopant is adsorbing. Erwin et al. [37] calculated the binding energies for Mn adsorbates on the surfaces of various semiconductors. They found that the binding energies of Mn on the (0001) surfaces of CdS and CdSe with wurtzite structures were higher than on the $\left(11\bar{2}0\right)$ or $\left(10\bar{1}0\right)$ surfaces. The adsorption energies of the dopant affect the doping efficiency and through which facet the doping will preferentially occur. Furthermore, dopant incorporation causes additional changes to the energy of the facets, and the adsorption of the dopant species on certain surfaces also modifies their reactivity. The latter effect can account for the observation that many ionic species are able to control the morphology of nanostructures without being incorporated into the host structure [21,39,40,41]. Consequently, the growth rates of the different facets will be different. It is inferred from this discussion that a possible “side effect” of the different adsorption energies of the dopants on different surfaces is the anisotropic growth of the nanocrystals, as observed in this work, which will depend on the concentration of the dopant. The assembly of particles in solution is usually promoted by intermolecular interactions established between molecules capping the particles, which are added to the reaction mixture or, in special cases, are formed in situ [12,15,42]. The syntheses of the Co-doped ZnO materials were performed in the absence of coordinating molecules such as surfactants. However, it has been found that oxidation of benzyl alcohol at high temperatures (ca. >230 °C) results in the formation of high amounts of benzoate species attached to the metal oxide nanoparticles, which can promote the assembly of particles and formation of supercrystals by π–π interactions between the aromatic rings [42]. The amount of benzoate ligands attached to the surface of doped zirconia nanocrystals was found to increase with the reaction temperature and with the amount of dopant [42]. The FTIR spectra of the ZnO and Co-doped ZnO nanostructures (Figure S2) do not indicate the presence of benzoate species. The spectra shows two bands at 1583 and 1416 cm^−1^, attributed to the antisymmetric and symmetric stretching vibration modes of the coordinated carboxylate moiety, but no bands from the vibration modes of aromatic rings are present. This suggests that the particles contain acetate ligands from the precursors adsorbed on the surface. However, carbon elemental analysis revealed that the amount of organics adsorbed is relatively small for promoting the aggregation of the particles (C wt % between 2.5 and 3.5), and that there is no correlation between the amount of dopant (and, therefore, the tendency to assemble) and the amount of organic species on the material. The aggregation process seems to be associated with the amount of cobalt in the synthesis. A possible explanation for the effect of cobalt on the assembly process is that cobalt ions adsorbed on the surface of the nanocrystals act as bridges between the nanoparticles during the early growth stages, promoting their assembly.

**Figure 2:**
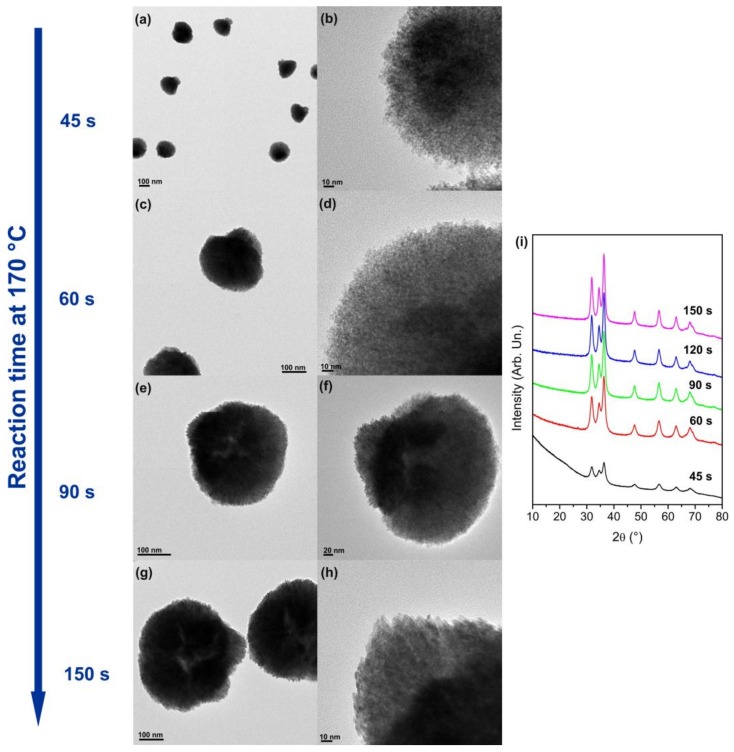
(**a**–**h**) TEM images and (**i**) XRD patterns of the products obtained at different reaction times during the synthesis of Co_0.15_Zn_0.85_O.

To gain insights into the evolution of the morphology and assembly of the nanoparticles, the formation reaction of Co_0.15_Zn_0.85_O was followed by TEM and XRD, as described in Section 2. For that purpose, the microwave (MW) reaction was stopped after just 30 s, 45 s, 60 s, 90 s, 120 s, and 150 s at 170 °C, and the solid product was characterized. The results are shown in Figure 2. No solid product was present after only 30 s of reaction, likely because at that point the mixture contained only monomeric species. On the contrary, agglomerates of nanoparticles are already formed after 45 s, suggesting that the assembly process occurs during the nucleation or early growth stages of particle formation. These agglomerates have a bouquet-like morphology and are made of very small nanoparticles of 2–3 nm with near spherical shape. The XRD indicates that the nanoparticles are already crystalline with wurtzite structure. Furthermore, the Co/Zn ratio in the agglomerates is similar to that of the final product (after 5 min reaction). Considering that the nanoparticles at this stage have near spherical morphology similarly to the undoped ZnO material and the Co precursor is slightly less reactive than the zinc precursor, it is likely that part of the Co is still adsorbed at the surface, possibly bridging neighboring nanoparticles, which leads to the formation of the assemblies at the nucleation/early growth stage. At 60 s, the size of the assemblies increase to ca. 250 nm. After 90 s of reaction, in addition to a further increase in the size of the aggregates to 250–300 nm, changes in the shape of the nanoparticles are observed. Most of the nanoparticles have irregular shape with sizes between 5 and 8 nm, but in some parts of the assemblies triangular NPs are already seen. For longer reaction times, the assemblies do not grow much more and the main change observed is the modification of the shape of individual nanoparticles. Therefore, these results confirm that the cobalt dopant controls the morphology of the nanoparticles during their growth. On the one hand, the preferential adsorption of cobalt on specific surfaces can hinder the growth of those facets and consequently the growth is promoted along other directions. On the other hand, the preferential incorporation of Co on specific surfaces changes the energy of those facets and consequently change the dissolution rate of the different facets promoting the growth in specific directions during Ostwald ripening, which is the growth mechanism of the ZnO nanoparticles synthesized by the reaction studied here [33]. After 150 s of reaction, most of the particles in the assemblies have triangular shape and the product is similar to the one obtained after 5 min of reaction.

**Figure 3:**
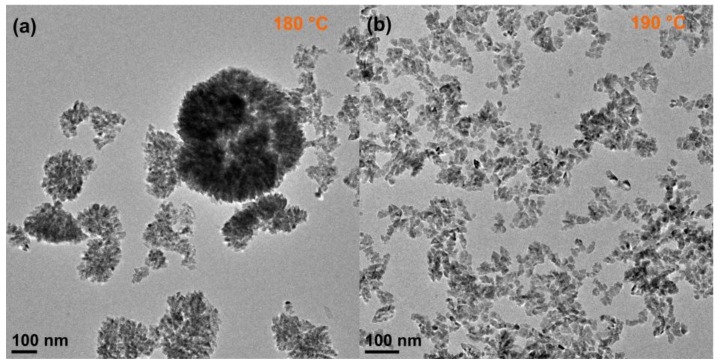
TEM images of Co_0.15_Zn_0.85_O synthesized at (**a**) 180 °C and (**b**) 190 °C.

Bilecka et al. [29] have previously reported the synthesis of Co_0.15_Zn_0.85_O nanoparticles also by reaction of Co and Zn acetates with benzyl alcohol under microwave irradiation, although at lower temperature (160 °C) and for shorter reaction time (3 min). Interestingly, the Co_0.15_Zn_0.85_O nanomaterial consisted of non-assembled nanoparticles with irregular shape. The difference between those and our results suggests a strong dependence of the morphology of this material and assembly behavior on the reaction temperature. Therefore, the synthesis of Co_0.15_Zn_0.85_O was additionally performed at the temperatures of 180 °C and 190 °C. Representative TEM images are shown in Figure 3. Increasing the reaction temperature by only 10 °C results is the formation of a mixture of nanoparticles with irregular and triangular shapes; part of the particles are aggregated in ill-defined structures. At 190 °C, the nanoparticles have irregular morphology and are not assembled. These results show that the promotion of the assembly of the nanoparticles and the changes in morphology are affected not only by the amount of cobalt present in the reaction mixture but also by the temperature. This is understood considering the effect of the temperature on the reactivity of the precursors, the surface adsorption energies, and nucleation/growth rates. As reported by Chen et al. [38], the doping of nanocrystals can involve several separated processes such as surface adsorption and lattice incorporation that show strong dependence on the temperature. Therefore, it seems that 170 °C is the optimal reaction temperature, at which the balance between the reactivity of the precursors, surface adsorption energies, cobalt incorporation rate and nanocrystal growth rate is the ideal for promoting the assembly of the nanoparticles from an early stage of the growth process and directing the shape of the nanoparticles into a triangular one.

## 4. Conclusions

Co_x_Zn_1−x_O nanomaterials with *x* = 0.05, 0.09, and 0.15 were synthesized by non-aqueous sol-gel in benzyl alcohol at 170 °C with microwave heating. The dopant was found to have a strong impact on the morphology of the nanostructures, which was attributed to the modification of the energies of the different facets, caused by the dopant adsorption and incorporation, that promoted the growth in preferential directions. Undoped ZnO consists of quasi-spherical nanoparticles, whereas Co_0.15_Zn_0.85_O is made of triangular nanoparticles assembled into spherical structures. The assembly possibly results from surface adsorbed cobalt species that bridge adjacent nanoparticles during the early growth stages of the particles. The morphology and assembly behavior of the Co_0.15_Zn_0.85_O nanoparticles are sensitive to the reaction temperature. In the temperature range studied (170–190 °C), the formation of well-defined assemblies of triangular nanoparticles was only observed at 170 °C, suggesting that the assembly process requires the correct balance between several processes that occur in solution during the nanostructure formation (e.g., surface adsorption energies, cobalt incorporation rate, and nanocrystal growth rate), which are differently affected by the temperature.

## Supplementary Materials

The following are available online at http://www.mdpi.com/2079-4991/8/4/249/s1, Figure S1: EDX spectra of the Co-doped ZnO materials, Figure S2: FTIR spectrum of Co_0.15_Zn_0.85_O.
