# Supplementary material for: Cobalt-Assisted Morphology and Assembly Control of Co-Doped ZnO Nanoparticles
Source: Nanomaterials (Basel). 2018 Apr 17;8(4):249. doi: 10.3390/nano8040249 (PMC5923579; doi:10.3390/nano8040249)
Supplement: Supplementary file 1 [file nanomaterials-08-00249-s001.pdf]

Cobalt-Assisted Morphology and Assembly Control of Co-Doped ZnO Nanoparticles

Xianying Han, Sebastian Wahl, Patrícia A. Russo * and Nicola Pinna *

Institut für Chemie and IRIS Adlershof, Humboldt-Universität zu Berlin, Brook-Taylor-Str. 2, 12489 Berlin, Germany; hanxiany@hu-berlin.de (X.H.); sebastian.wahl@chemie.hu-berlin.de (S.W.)

* Correspondence: patricia.russo@hu-berlin.de (P.A.R.); nicola.pinna@hu-berlin.de (N.P.)

Supplementary material

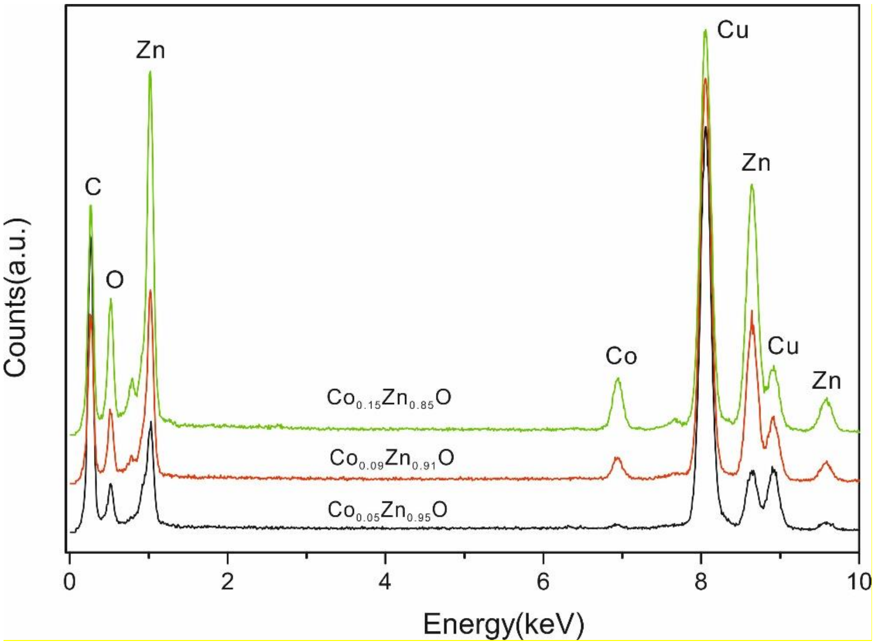

Figure S1. EDX spectra of the Co-doped ZnO materials.

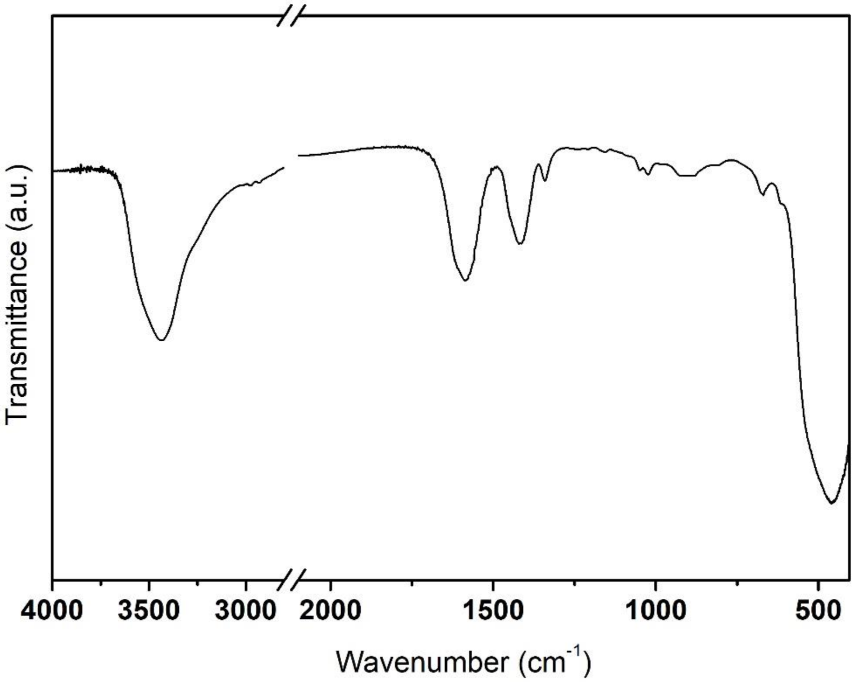

Figure S2. FTIR spectrum of $Co_{0.15}Zn_{0.85}O$.
